# Investigating the Underlying Mechanisms of Circular RNAs and Their Application in Clinical Research of Cervical Cancer

**DOI:** 10.3389/fgene.2021.653051

**Published:** 2021-03-25

**Authors:** Jian Liu, He Zhu, Li Fu, Tianmin Xu

**Affiliations:** Department of Gynaecology and Obstetrics, The Second Hospital of Jilin University, Changchun, China

**Keywords:** cervical cancer, circRNAs, precision medicine, molecular marker, miRNAs sponges

## Abstract

Circular RNAs (circRNAs) are non-coding RNA molecules, and these are differentially expressed in various diseases, including cancer, suggesting that circRNAs can regulate certain diseases. CircRNAs can act as miRNAs sponges, RNA-binding protein (RBP) sponges, and translation regulators, and they can become an important part of the regulation of gene expression. Furthermore, because of their biomedical features in body fluids, such as high abundance, conservation, and stability, circRNAs are seen as potential biomarkers for various cancers. Cervical cancer (CC) is one of the main causes of cancer-related death in women, and there have been a large number of studies that analyze circRNAs as a new object to be evaluated in CC. Therefore, this review, by understanding the role of circRNAs in CC, may create innovative strategies in the future clinical diagnosis, treatment, and prognosis of CC and promote the development of personalized and highly accurate cancer therapy.

## Introduction

**FIGURE 1:**
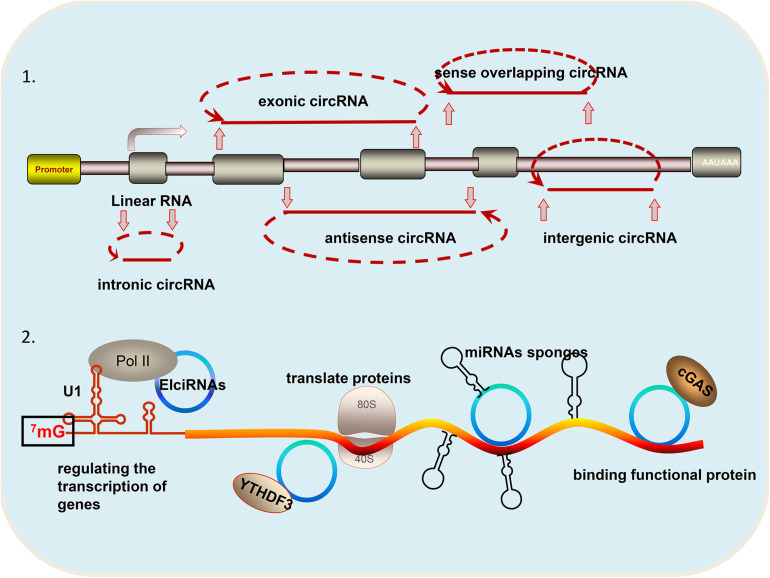
Different types and functions of circRNAs: (1) According to the source of circRNA, it can be divided into the following categories: circular intronic RNAs (ciRNA) derived from gene introns; exonic circRNA (ecircRNAs) derived from gene exons; intergenic circRNAs derived from breakpoints between known genes; antisense circRNA derived from the antisense strand of a known gene; and sense overlapping circRNA: derived from the same gene locus as the linear transcript. (2) CircRNAs have the following four functions: regulating the transcription of genes; translating proteins; acting as miRNA sponges; and binding functional proteins.

Circular RNAs (circRNAs) are a special class of non-coding RNA molecules that do not have a 5′ terminus cap and a 3′ terminus poly (A) tail. Unlike traditional linear RNA, circRNAs exhibit closed ring structures, are not affected by exonuclease RNaseR, and are more stable in expression. CircRNAs are mainly generated from gene exons, but there are other types, such as those from introns and those that are inter genic, antisense, and sense overlapping (Figure 1). Owing to *trans-*shear, circRNA is abundant in the cytoplasm of eukaryotic cells, and its localization in the cytoplasm is a sufficient and necessary condition to study the mechanism of miRNA sponge. Small numbers of intron-derived circRNAs are noted in nucleic acids and have certain tissue, timing, and disease specificities, and they are thus suitable for molecular markers (Zhang Z. et al., 2018). CircRNAs have become a research hotspot because they are closely related to the progress of disease.

## CircRNA Mechanisms of Action

There has been much research on circRNAs. They can be used as competing endogenous RNA (ceRNA) to regulate the physiological activities of cells; some circRNAs located in the nucleus can regulate the transcription of parental genes by binding to RNA

polymerase II; they can also bind to proteins, affecting the cell cycle; and they can even encode proteins like mRNA. Especially in recent years in the field of cancer research, this has opened up a fresh direction for circRNAs (Zhou et al., 2018). Several cancer studies have found dysregulation of circRNA expression in *in vitro* and *in vivo* trials, in clinical cancer sample tissues, and even in patients’ body fluid samples, which are associated with clinical features of cancers (Huang et al., 2017), such as reproduction and gynecological diseases (Liu et al., 2019), cancer of the digestive system (Sheng et al., 2018), glioblastoma (Zhang et al., 2017; Yang et al., 2018), drug resistance (Shao et al., 2018), and so on. The unique role of CircRNAs in carcinogenesis may provide potential targets for cancer therapy and may also inhibit or regulate the malignant behavior of cancer cells through novel transcriptional therapies (Zhao and Shen, 2017).

### CircRNA Acting as miRNA Sponge

Nikolaus Rajewsky first discovered antisense circRNA with cerebellar degeneration-related protein 1 transcript (CDR1as), which can be combined with miR-7 in neural tissue to prove that circRNA can act as a sponge (called ciRS-7) (Memczak et al., 2013). The data suggest that circRNA may act as a post-transcriptional regulator to competitively inhibit other RNAs from binding to miRNA and RBPs. It can often have a role in regulating local free concentrations of RBP, RNA, or their binding sites. While circRNA can completely resist the instability of miRNA target genes, it significantly inhibits miR-7 function, leading to elevated miR-7 target levels. Other studies have shown that sex-determining region Y (Sry), a kind of testis-specific circRNA, is an miR-138 sponge, indicating that some circRNAs contain response elements of miRNA that can act as ceRNA, thereby relieving miRNA inhibition of target genes and upregulating their expression (Hansen et al., 2013). High expression of circRNA_104075 in human hepatocellular carcinoma (HCC) cell lines adsorbs miR-582-3p of targeted inhibition of YAP expression through the sponge mechanism and promotes HCC occurrence and progression. It also reveals that circ_104075 has up to 96% sensitivity and 98.3% specificity in diagnostic HCC and has potential as a biomarker for diagnosis (Zhang X. et al., 2018).

### CircRNA Regulating Gene Expression

To further understand the function of circRNA, CDR1as was further studied. The authors selected four regions of the high expression of brain CDR1as in CDR1as mice and CDR1as-knockout (KO) mice for mRNA sequencing. Many of the genes identified as miR-7 targets showed significant changes. Consistent with changes in gene expression, mutant mice did exhibit abnormal neuronal activity in a series of *in vivo* behavioral experiments, suggesting circRNA plays a critical role in mouse behavior (Piwecka et al., 2017). Exon2, an abundant characterizing circRNA, derived from HIPK3, is called circHIPK3, and that silencing, rather than HIPK3 mRNA silencing, considerably inhibits human cell proliferation. Through a luciferase screening assay, circHIPK3 was noted to directly combine with miR-124 and then suppress miR-124 activity, suggesting that circRNA generated by precursor mRNA can regulate cell behavior (Zheng et al., 2016).

Functions of two circRNAs (circBIRC6 and circCORO1C) are related to pluripotent states, demonstrating that circRNAs also have the function of regulating human pluripotency, identifying subsets of circRNA enriched in human embryonic stem cells (hESCs). They also promote a pluripotent state by inhibiting their mediated reduction of NANOG and OCT4 expression and then inhibiting hESC differentiation through sponge mechanisms that bind directly to miR-34a and miR-145 (Yu et al., 2017). Unlike this, circCSNK1G3 and miR-181b/d in prostate cancer have synergies. Overexpression of miR-181b/d in PC-3 cell lines significantly decreased the abundance of tumor suppressor CBX7 in prostate cancer, upregulated cell cycle genes such as CDK1, CDC25A, increased the proliferation of PC-3 cells and, more importantly, attenuated circCSNK1G3 knockdown induced cell proliferation arrest. Taken together, circCSNK1G3 promotes the proliferation of prostate cancer cells through interactions with miR-181b/d, and this new regulation differs from the sponge mechanism (Chen S. J. et al., 2019).

### CircRNA Binding Functional Protein

Circular RNA can not only bind to the miRNA to play the role of a sponge, but it can also bind to functional proteins. One study explored circANRIL binding proteins through RNA pull down, and it was reverse confirmed by RNA binding protein immunoprecipitation (RIP) that circRNA was involved in the maturation process by binding to PES1 proteins (Holdt et al., 2016). Sub-populations of hematopoietic stem cells (HSEs) were significantly altered only after interfering with circRNA-Cia-cGAS transcribed by D430042O09Rik. The long-term hematopoietic stem cells (LT-HSC) decreased significantly, and the expression of type I interferon (IFN) increased in cia-cGAS KO mice. Enzymatic studies have demonstrated that cia-cGAS in the nucleus inhibits its enzyme activity by binding DNA-sensitive cGAMP synthetase (cGAS) and then preventing the resting LT-HSC from being cGAS depleted. Additionally, cia-cGAS has a stronger affinity to cGAS compared with linear cGAS and thereby inhibits the generation of type I IFN in LT-HSCs, which was mediated by cGAS. These experiments found a novel circRNA—cia-cGAS that binds cGAS and inhibits its activity, regulates the LT-HSC resting state, and reduces energy depletion and apoptosis caused by type I IFN (Xia et al., 2018).

### CircRNA Regulating the Transcription of Genes

Circular RNAs can regulate transcription through specific RNA-RNA interactions and then regulate source gene expression. Through CLIP-seq, researchers found that RNA Polymerase II incorporated some circRNAs, formed by introns, between exons and retained exons named exon-intron circRNA (EIciRNAs), such as circEIF3J and circPAIP. By fluorescence *in situ* hybridization (FISH) localization, the researchers concluded that it was located in the nucleus and could regulate the expression of the genes from which it originated. Experiments then showed that EIciRNAs co-conjugated with Pol II, U1, and small nuclear ribonucleoproteins (snRNP) at 300 bps upstream of the transcription start site of the gene from which it originated and also that the binding with U1 and snRNP was necessary (Li et al., 2015).

### CircRNA Translating Proteins

Originally, we thought that circRNAs belonging to non-coding RNA could not play the function of coding proteins; Yun Y. et al., proved that circRNA can translate proteins, but most circRNAs are non-coding RNAs. CircRNA does not have a free 5′ and 3′ terminus; if it can be translated, it must thus be done in a way that does not depend on the 5′ cap structure, such as through the internal ribosome entry site (IRES) or modified N6-methyladenosine (m6A) modification. The study detected no IRES on the circRNA, but it can also translate proteins. A series of experiments confirmed that the recognition proteins YTHDF3 recognized the m6A modification occurring on the circRNA and recruited translation initiation factors such as eIF3A and eIF4G2 (Yang et al., 2017). The circRNAs are widely m6A modified and present cell-specific features. The m6A modification enzyme of circRNA is consistent with the mRNA, while the m6A modification sites are different (Zhou et al., 2017). Moreover, circRNA-encoded proteins are functional, such as FBXW7-185aa, a new 21 kDa protein, which was encoded by circ-FBXW7. Upregulation of FBXW7-185aa inhibits cell proliferation but accelerates the cell cycle, whereas knockdown of FBXW7-185aa promotes the malignant behavior of cells *in vivo* and *in vitro*. Furthermore, circ-FBXW7 expression is correlated with the prognosis of glioblastoma patients (Yang et al., 2018).

## Building CircRNA Related Information Networks in Cervical Cancer

### Building Networks Through Bioinformatics Data

Through the use of the Molecular Complex Detection building protein–protein interaction (PPI) network to analyze the comprehensive bioinformatics of cervical cancer, seven central genes were identified (RRM2, CEP55, CHEK1, KIF23, RACGAP1, ATAD2, and KIF11). A circRNA-miRNA-mRNA network was constructed that included five circRNAs (hsa_circRNA_101958, hsa_circRNA_400068, hsa_circRNA_103519, hsa_circRNA_104315, and hsa_circRNA_000596), two mRNAs (hsa-miR-106b and hsa-miR-15b), and seven mRNA corresponding to the seven central genes, and 22 circRNA-miRNA-mRNA control axes were determined in the sub-net (Yi et al., 2019). Similarly, another study also constructed ceRNA networks for CC through Gene Expression Omnibus (GEO) database and the Cancer Genome Atlas (CGA) database, used the STRING database to analyze protein interactions, and used the MCODE plugin to identify hub genes (Gong et al., 2019). These results can provide some insight for future research on the molecular mechanism of CC, but since the research is based on computer data, further experiments are necessary in order to verify it.

### Building Networks Through High-Throughput RNA Sequencing (RNA-Seq)

Tumor tissues and adjacent non-tumor tissue (ANT) tissues were studied in patients with cervical squamous cell carcinoma (CSCC) by RNA-seq. It identified 19 lncRNAs, 99 circRNAs, and 28 miRNAs; 304 mRNAs were differentially expressed (DE); and it constructed ceRNA networks of coded and non-coding RNA to predict interactions between lncRNA, circRNA, miRNA, and mRNA. The findings revealed for the first time that circRNA may be involved in CC and that ceRNA of DE may develop further as biomarkers (Wang H. et al., 2017).

Furthermore, through RNA-seq, the DE of circRNA between radiated CC cells and non-radiated CC cells was studied, and cytoscape-mapped circRNA–miRNA–target gene interaction networks were described. Studies revealed that, through different mechanisms, radiotherapy can kill HeLa cells and promote cell migration and invasion. The Kyoto Encyclopedia of Genes and Genomes (KEGG) and Gene Ontology (GO) analysis indicated that the MAPK signal transduction pathway is the most abundant pathway, and high expression of target genes may be related to angiogenesis and cell metastasis. The exploration of circRNA associated with radiation resistance in HeLa cells of radiotherapy may be used to provide appropriate treatment strategies and improve prognosis in resistant cancers (Yu et al., 2018).

### Network Constructions via Microarray

Bioinformatic prediction and the widespread application of RNA-seq technology have identified a large number of circRNAs. However, at present, to fully characterize these circRNAs we require samples of interest, which we currently lack. Microarray detection may show effective detection efficiency. One study provides abundant resources from 41 microarray datasets, providing strong evidence for the expression of circRNA in CC (Li et al., 2018). Using high-throughput microarray technology, the expression of circRNAs was also studied by transfecting E7 siRNA in HPV16-positive CaSki cells. Eight of them are strongly expressed, the expression of hsa_circ_0026527, hsa_circ_0056353, hsa_circ_0035918, hsa_circ_0037213, hsa_circ_0038475, and hsa_circ_0048867 was downregulated, and the expression of hsa_circ_0052602 and hsa_circ_0051620 was upregulated. Further analysis clarified through the GO and KEGG databases showed that several circRNAs can be used as ceRNAs to regulate the occurrence and development of CC, and DE of circRNAs may be related to the proline metabolism, glutathione metabolism, and the mTOR signaling pathway (Zheng et al., 2018). The miRNA target of circRNA was predicted by TargetScan Human and miR Base, but the point of whether it was directly directed to sponge circRNAs or targeted to regulate its expression to cause tumorigenesis was not made.

## Mechanism of CircRNA in Cervical Cancer

**FIGURE 2:**
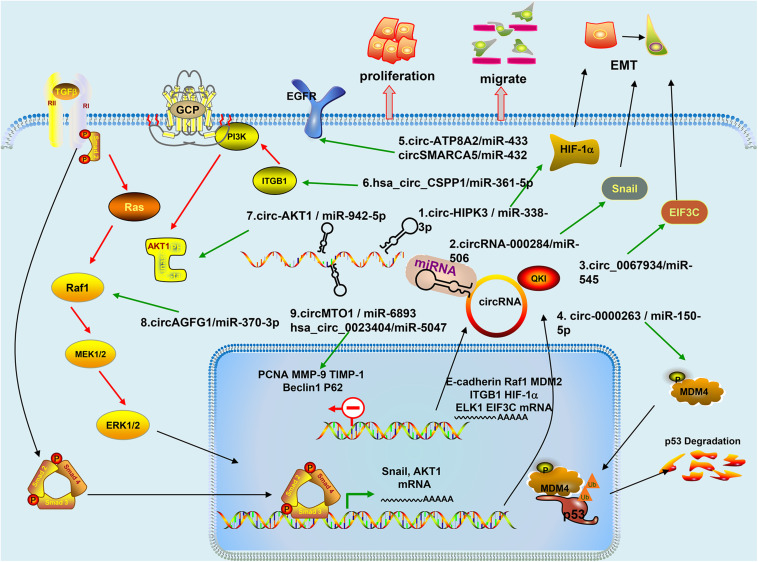
Mechanism of circRNAs in Cervical Cancer: (1) Circ-HIPK3 acts as a competing endogenous RNA of miR-338-3p, via regulating HIF-1α mediated EMT; (2) CircRNA-000284 suppresses the expression of Snail-2 via sponging miR-506; (3) Circ_0067934 modulates EIF3C by sponging miR-545; (4) Circ-0000263, as a competitive endogenous RNA, regulates MDM4 expression by sponging miR-150-5p and may play an important role affecting the expression of p53 gene; (5) Circ-ATP8A2 sponges miR-433 to release its suppression on EGFR expression. CircSMARCA5 modulates miR-432 via the ERK signaling pathway; (6) Hsa_circ_CSPP1 regulates miR-361-5p/ITGB1 in the PI3K-Akt signaling pathway; (7) CircRNA-AKT1 sequesters miR-942-5p to upregulate AKT1; (8) CircAGFG1 enhances the activity of RAF/MEK/ERK pathway by sponging miR-370-3p and further regulating RAF1; and (9) CircMTO1 directly interacts with miR-6893 and their inhibitor enhanced Beclin 1 expression and downregulated p62 level. Hsa_circ_0023404 knockdown can also promote p62 protein levels and attenuate Beclin 1 level.

Cervical cancer is the most common gynecologic tumor and can provoke a large number of deaths every year. For the most part, a high-risk subtype of human papillomavirus (HPV) is in charge of the disease, which is largely preventable (Cohen et al., 2019). CircRNAs are the focus of current research, and several studies have speculated on the role of circRNAs and inferred their potential mechanisms in CC (Chaichian et al., 2019). A series of studies have proven the effects of circRNA on CC cell lines in *in vitro* and *in vivo* experiments (Table 1); further studies have indicated that circRNA is involved in the progression of cervical tumors through a variety of mechanisms (Figure 2), with miRNA sponges as the main mechanism, and they have even shown that circE7 produces E7 proteins by m6A modification (Zhao et al., 2019). We reviewed some studies conducted so far to explore the functions of circRNAs in the occurrence and development of CC and the relationship between circRNAs and metastasis, invasion, recurrence, and chemical resistance of cervical cancer.

**TABLE 1:** The effects of circRNA on CC cell lines.

circRNA	miRNA	CC cell lines and Xenograft	Function	Type of detection methods	References
circ-0033550 (circRNA-AKT1)	miR-942-5p	HeLa, CaSki, SiHa, C33A, 293T, and H8 cell lines; Male BALB/c nude mice	Promotes cell proliferation and invasion; Increased the volume and weight of xenografts	CCK-8, transwell, colony formation, FISH, Immunofluorescence (IF) staining, dual-luciferase reporter, 5-Ethynyl-2′-deoxyuridine (EdU), RNA pull-down, and RIP assays	Ou et al., 2020b
circAGFG1	miR-370-3p	HeLa, C-33A, SiHa, HCC94, and End1/E6E7 (normal cervical cell line)	CircAGFG1 downregulation restrained cell viability, proliferation and migration, and promoted cell apoptosis	loss-of function, bioinformatics analysis, mechanism experiments, and rescue assays	Wu and Zhou, 2019
circular RNA hsa_circ_0000515	miR-326	Hela and SiHa cell lines; Clean grade female BALB/c nude mice	Hsa_circ_0000515 silencing attenuated cell proliferation and invasion; promoted apoptosis and autophagy; and reduced tumor volume and weight	EdU, Monodansylcadaverine (MDC) staining, flow cytometric, transwell, FISH, RIP, dual-luciferase reporter, and TdT-mediated dUTP-biotin nick end-labeling (TUNEL) staining assays	Tang et al., 2019
circular RNA hsa_circ_0023404	miR-136	SiHa, C-33a, CaSki, C4-1 and Hela, and human cervical epithelial cells (CerEpiC)	Hsa_circ_0023404 knockdown significantly suppressed cell proliferation and colony formation	CCK-8, transwell, colony formation, cell cycle distribution, and luciferase reporter assays	Zhang Z. et al., 2018
circCLK3	miR-320a	SiHa, HeLa, CaSki, C-33A, MS751 cell lines; BALB/c athymic nude mice	Promotes cell proliferation, migration, and invasion. Promoted growth and metastasis of tumor *in vivo*	CCK-8, cell colony, wound healing, transwell migration and invasion, RNA pull-down, RIP, luciferase reporter, and rescue assays	Hong et al., 2019
circular RNA HIPK3	miR-338-3p	SiHa and C-4I cell lines	Circ-HIPK3 silencing inhibited cell proliferation, migration and invasion, while induced apoptosis	CCK-8, cell formation, cell apoptosis, transwell migration and invasion, point mutation, RNA pull-down, luciferase reporter, and rescue assays	Qian et al., 2020
circ_0067934	miR-545	SiHa, CaSki, Hela, and C4-1 and immortalized cervical epithelium NC104 cell line; BALB/c nude mice	Promotes proliferation, colony formation, migration, invasion, and epithelial-mesenchymal transition of CC cells. Promoted tumor growth *in vivo*	CCK-8, colony proliferation, transwell, and luciferase reporter assays	Hu et al., 2019
circEIF4G2	miR-218	HeLa, CasKi, C33A, and SiHa cells	CircEIF4G2 knockdown significantly inhibited cell migration and invasion	CCK-8, colony formation, wound healing, transwell migration and invasion, RIP, and dual-luciferase reporter assays	Mao et al., 2019
circSLC26A4 (hsa_circ_0132980)	miR-1287-5p	CaSki, SiHa cell lines; Male BALB/c nude mice	CircSLC26A4 silencing inhibited cell proliferation, invasion, and effectively repressed tumor growth *in vivo*	CCK-8, colony proliferation, transwell, RIP, dual-luciferase reporter, and RNA-FISH assays	Ji et al., 2020
circSMARCA5	miR-620	HeLa, CaSki, SiHa, C33 cell lines, and 293T cells	CircSMARCA5 dramatically inhibited the proliferation and colony-forming abilities of CC cells	Luciferase activity, MTT, and cell invasion assays	Dai et al., 2018
circ-ATP8A2	miR-433	HeLa and SW756 cell lines	Circ-ATP8A2 knockdown inhibited cell proliferation, migratory and invasive capacities and increased apoptotic cells	CCK-8, acridine orange/ethidium bromide (AO/EB), flow cytometric, transwell, and dual-luciferase reporter assays	Ding and Zhang, 2019
circMTO1	miR-6893	HeLa, CaSki, C-33A, C-4 II, SiHa and immortalized epithelial cells of human ectocervix Ect1/E6E7 cell lines; NOD-SCID immunodeficient mice	CircMTO1 knockdown suppressed cell migration, invasion, chemoresistance and markedly impaired tumor growth ability *in vivo*	Wound healing, transwell invasion, MTT, TUNEL, and luciferase reporter assays	Chen M. et al., 2019
circRNA hsa_circ_0023404	miR-5047	HeLa, SiHa cell lines and 293T cell	Knockdown in HeLa and SiHa cells reduced the number of invaded cells;promotes metastasis of Human Dermal Lymphatic Endothelial Cells (HDLEC)	Transwell invasion, lymphatic vessel, MTT, flow cytometry analysis, and luciferase reporter assays	Guo et al., 2019
circ_0005576	miR-153	HeLa, SiHa, Caski, C-33A and immortalized cervical epithelium cell lines (HcerEpiC)	Promotes cell proliferation, migration, and invasion	CCK8, colony formation, transwell, RNA pull-down, luciferase reporter, and RIP assays	Ma et al., 2019
hsa_circ_0018289	miR-497	HeLa, CaSki, SiHa, HT-3,C33A and human epidermal cell (HaCaT); Male BALB/c nude mice	Hsa_circ_0018289 knockdown inhibited cell proliferation, migration and invasion. Decreased the tumor volumes and weights	CCK8, transwell, and luciferase reporter assays	Gao et al., 2017
circRNA-000284	miR-506	HeLa, CaSki, SiHa, C-33A, and SW756 cell lines	Promotes cell proliferation and invasion	CCK8, transwell, dual-luciferase reporter, and RNA-FISH assays	Ma et al., 2018
circular RNA hsa_circ_0000263	miR-150-5p	HeLa, CaSki, SiHa, C-33A, and SW756 cell lines	Hsa_circ_0000263 downregulation inhibited cell proliferation and migration; promoted cell apoptosis	CCK-8, cell proliferation, transwell, flow cytometric, RIP, and dual-luciferase reporter assays	Cai et al., 2019
hsa_circ_0007534	miR-498	HeLa, SiHa, and CaSki, and human cervical epithelial immortalized cell line H8	Hsa_circ_0007534 inhibition impeded cell proliferation and invasion	CCK-8, colony proliferation, transwell, RNA pull-down, and dual-luciferase reporter assays	Rong et al., 2019
circAMOTL1	miR-485-5p	HeLa, CaSki cell lines; BALB/c nude mice (C-33A cells)	Induced cell proliferation and migration. Promoted cervical cancer development *in vivo*	CCK-8, wound healing, transwell, EdU incorporation, RNA pull-down, FISH, and luciferase reporter assays	Ou et al., 2020a
circ-ITCH	miR-93-5p	SiHa, Caski, HeLa, and C33A cell lines; SPF grade BALB/c nude mice	Inhibited cell proliferation, migration and invasion. Significantly inhibited tumor growth *in vivo*	CCK-8, colony proliferation, transwell, luciferase reporter, and tumor xenograft assays	Li et al., 2020
circ-MYBL2	miR-361-3p	HeLa, CaSki cell lines	Circ-MYBL2 inhibition suppressed cell proliferation and invasion	CCK-8, colony formation, transwell invasion, RNA pull-down, and luciferase reporter assays	Wang J. et al., 2019
hsa_circ_0075341	miR-149-5p	SiHa and CaSki cell lines	Promotes cell proliferation and invasion	CCK-8, colony formation, transwell invasion, and dual-luciferase reporter assays	Shao et al., 2020
hsa_circ_0001038	miR-337-3p	HeLa, SiHa, C-33A, SW756, and normal cells (HcerEpiC)	Hsa_circ_0001038 knockdown inhibited cell proliferation, migration and invasion	CCK-8, flow cytometric, transwell, AO/EB staining, loss/gain-of function, and dual-luciferase reporter assays	Wang Y. et al., 2020
circRNA8924	miR-518d-5p/519-5p	SiHa and HeLa cell lines and 293T cells	CircRNA8924 knockdown significantly inhibited cell proliferation, migration and invasion	CCK-8, flow cytometric, transwell, loss/gain-of function, and luciferase reporter assays	Liu et al., 2018
circ_0000388	miR-337-3p	HeLa and SiHa cell lines	Notably enhances cell migration and invasion	CCK-8, TUNEL, wound healing, transwell, RT-PCR, Western blot, RIP, and luciferase reporter assays	Meng et al., 2020
circ_103973	miR-335	HeLa, CaSki, C33A, and SiHa cell lines	Circ_103973 knockdown promoted cell apoptosis and inhibited cell proliferation	Flow cytometric, MTT, colony formation, dual-luciferase reporter, and RNA pull-down assays	Zhu et al., 2020
circRNA_0000285	–	HeLa, SiHa, C4-1, and C-33A and normal cervical epithelium cell line (NC104); NOD/SCID mice	CircRNA_0000285 knockdown inhibited cell proliferation and invasion; inhibited tumor formation and metastasis	CCK-8, cell cycle, and transwell assays	Chen R. X. et al., 2019
Hsa_circRNA_101996	miR-8075	SiHa, C33A, CaSki, and Hela cell lines; BALB/c nude mice	Promotes cell proliferation, migration, and invasion. Led to the decrease of the tumor size and weight	CCK-8, cell invasion, colony formation, and luciferase reporter assays	Song et al., 2019

### Participation in Related Signaling Pathways

#### Controlling PI3K-Akt Signaling Pathways

One study found that circ-0033550 was upregulated in CC, and its related gene was AKT1, and the circ-0033550 was thus renamed circ-AKT1. AKT1, as a serine/threonine kinase, has highly conserved properties and is a core node of the phosphatidylinositol 3-kinase (PI3K)/AKT pathway. AKT1 is involved in multiple biological manners, including metabolism, cell survival, and migration. The loss/gain-of-function assays showed that circ-AKT1 regulated AKT1 and then promoted cell proliferation and invasion in CC *in vitro*, and it was verified that the tumor growth was promoted in CC by *vivo* assays. Mechanically, circ-AKT1 raised AKT1 through sponging miR-942-5p. Moreover, transforming growth factor-beta (TGF-β) induces circ-AKT1 and AKT1. All in all, circRNA-AKT1/miR-942-5p upregulated AKT1 and then facilitated CC tumorigenesis. The circ-AKT1/miR-942-5p/AKT1 axis may provide novel molecular targets for therapeutic improvement in CC (Ou et al., 2020b). In other studies, hsa_circ_CSPP1 can regulate the proliferation and migration of CC cells through the miR-361-5p/ITGB1 axis, which is a part of the PI3K/AKT pathway. Hsa_circ_CSPP1 acts as a sponge of miR-361-5p, and, meanwhile, miR-361-5p, by downregulating ITGB1, inhibits cell activity and mobility and induces cell apoptosis, thereby inhibiting cervical cancer. Thus, inhibiting hsa_circ_CSPP1 and then inhibiting the expression of downstream genes such as PI3K, AKT, and ITGB1 prevents tumor growth (Yang and Xie, 2020).

#### Involvement in MAPK Signaling Pathways

CircAGFG1 was upregulated in CC cell lines *in vitro* and, the decrease in circAGFG1 levels was proven to inhibit the proliferation and migration ability of CC cells. CircAGFG1 depleted miR-370-3p, which regulates RAF1 expression, and then activated RAF/MEK/ERK pathways through bioinformatics analyses and further experiments. Thus, circAGFG1 can influence the proliferation and migration of CC cells through miR-370-3p/RAF1 (Wu and Zhou, 2019). The overexpression of hsa_circ_0000515 in cervical cancer samples was identified by microarray data analysis (GSE102686), and it was predicted that hsa_circ_0000515 directly regulated miR-326 using a biological database and RT-qPCR. Further experimental results emphasized that hsa_circ_0000515 can be used as an miR-326 ceRNA to increase ELK1 expression. Furthermore, enhancing ELKI expression leads to increase proliferation and invasion but inhibits apoptosis and autophagy of CC cells. Experiments *in vivo* further confirmed that hsa_circ_0000515 silencing inhibited tumor growth. ELK1 is the integration point of other pathways associated with mitogen-activated protein kinase (MAPK) signal pathways, and we may thus infer that potential MAPK pathways may be highly likely involved in hsa_circ_0000515/miR-326/ELK1 axis (Tang et al., 2019). Some findings also provide a hypothesis that the mechanism of hsa_circ_0101996 involved in the progression of CC may be achieved by activating MAPK signaling (Wang Y.-M. et al., 2017).

#### Involvement in Hippo Signaling Pathways

YAP1 is the transcription effector of the Hippo signaling pathway and mediates the activity of stem cell and tissue expansion through interactions with TEAD transcription factors (Schlegelmilch et al., 2011). One study has shown that hsa_circ_0023404 regulated TFCP2 via sponging miR-136, and TFCP2 is an activator of YAP pathways. Experiments proved that hsa_circ_0023404 activated the Hippo pathway by stimulating miR-136, promoting the expression of TFCP2 in CC, which leads to the development and progression of CC. The study uncovered a novel therapeutic ring in the process of cervical cancer, which is hsa_circ_0023404/miR-136/TFCP2/YAP axis (Zhang J. et al., 2018).

### Regulation of Relating Genes Expression Through Sponge Mechanisms

#### Mediating Epithelial-Mesenchymal Transition (EMT)

The downregulation of E-cad is generally considered to be a result of EMT and is recognized as a key link promoting progression and metastasis of cancer. In SiHa and CaSki cells, the low expression level of circ-0745 decreased cell proliferation, invasion, and migration *in vitro* and also reduced the tumor size *in vivo*. The knockdown of hsa_circ_0000745 can reduce the expression of E-cad, and the upregulation of circ-0745 can inhibit E-cad expression, thereby facilitating the transfer of cells and leading to the development of cancer (Jiao et al., 2020). Similarly, the circCLK3/miR-320a/FoxM1 axis also impacted the progression of CC through EMT. Overexpression of miR-320a inhibited the activity of circCLK3 to increase the expression of E-cadherin. MiR-320a targets FoxM1 increased E-cadherin expression but downregulated the expression of N-cadherin and Vimentin (Hong et al., 2019). In another study, TGF-β targets circ-AKT1/miR-942-5p/AKT1 axis and then promotes EMT of CC cells (Ou et al., 2020b). The expression of circ-HIPK3 in cervical cancer tissues was markedly upregulated compared to ANT. Through sponging miR-338-3p, circ-HIPK3 upregulated hypoxia-inducible factor-1α (HIF-1α). Overexpression of HIF-1α or miR-338-3p silencing can rescue suppression of CC malignant features caused by the knockdown of circ-HIPK3. Therefore, circ-HIPK3 as ceRNA of miR-338-3p regulates EMT mediated by HIF-1α and further promotes CC cell growth and metastasis. Targeting the circ-HIPK3/miR-338-3p/HIF-1α axis will be a new type of regulating strategy for CC (Qian et al., 2020). Circ_0067934 also facilitates cell proliferation, migration, and invasion, which was caused by EMT in CC *via* a series of assays. An RNA pull-down assay found that circ_0067934 precipitated miR-545 through using biotin-labeled circ_0067934 probes in SiHa and HeLa cells, which indicated direct interaction between circ_0067934 and miR-545. Further study showed that miR-545 inhibits cell proliferation and invasion, and the effect of circ_0067934 knockdown can be rescued by the recovery of EIF3C. This proves that circ_0067934 promotes CC development through miR-545/EIF3C axis (Hu et al., 2019). By culturing exo-circ_PVT1/exo-vector with C33A, the researchers found that circ_PVT1 induced the downregulation of E-cadherin and the upregulation of Vimentin, N-cadherin, and SNAIL by targeting miR-1286 (Wang H. et al., 2020). This suggests circ_PVT1 can induce EMT in CC cells through the exosome pathway, which is a new mechanism for the progression of cervical cancer.

#### Targeted HOXA Cluster Gene

CircEIF4G2 in cervical cancer tissues was strongly higher than that in matched ANT and was evidently related to tumor size, lymph node metastasis, and poor prognosis but not related to the stage of tumor lymph node metastasis or the patient’s age. Moreover, circEIF4G2 can sponge miR-218 to enhance the expression level of the target gene homeobox a1 (HOXA1). Transfection of cells with miR-218 inhibitors attenuated the suppression of malignant cell behavior due to circEIF4G2 knockdown. Silencing HOXA1 can reverse the effect of miR-218 inhibitors on Hela cells. These results show that circEIF4G2/miR-218/HOXA1 pathways promote tumor formation and metastasis (Mao et al., 2019). CircSLC26A4 and miR-1287-5p have a negative correlation function and are both located in the cytoplasm of CC cells, which is proven by RNA fluorescence *in situ* hybridization (RNA-FISH). Additionally, miR-1287-5p can combine with HOXA7 through their complementary binding domains. Therefore, circSLC26A4 may act as miRNA sponges to mediate CC tumor phenotypes (Ji et al., 2020).

#### Targeting Epidermal Growth Factor Receptor (EGFR)

CircSMARCA5 overexpression inhibits CC tumor phenotypes. The results of the study suggest that circSMARCA5 can target miR-432 that interacts with EGFR by binding to the 3′-non-translational region. Thus, circSMARCA5 may regulate miR-432 and then, through the ERK signaling pathways, act as a vital role in the progression of CC (Huang et al., 2020). Another finding found that circSMARCA5 can also bind to miR-620 and inhibit the proliferation and invasion of CC cells, and it is involved in the development of CC through regulating the circSMARCA5/miR-620 axis (Dai et al., 2018). Hence, targeting circSMARCA5 can be considered as an emerging therapeutic option for cervical cancer. Circ-ATP8A2 was detected in CC specimens and cells and interacted with miR-433. Immunoblotting analysis showed that the expression of EGFR decreased considerably after silencing circ-ATP8A2, while co-transfection with the miR-433 inhibitor or EGFR vector efficiently increased the expression level of EGFR. Circ-ATP8A2 released the inhibition of EGFR expression caused by miR-433 at the posttranscriptional level, i.e., circ-ATP8A2 can promote CC cell progression through the miR-433/EGFR axis (Ding and Zhang, 2019).

#### Regulation of Autophagy-Related Genes (ATG)

CircMTO1 can interact with miR-6893 and is significantly upregulated in CC cells in an *in vitro* experiment. Meanwhile, a xenograft tumor assay proved that miR-6893 inhibitors can save the tumorigenic effects of CC cells caused by circMTO1 knock-down. S100A1 was identified as the target for circMTO1 and miR-6893 induction tumorigenesis of CC. Western blot analysis showed that both circMTO1 and miR-6893 inhibitors can enhance Beclin 1 expression and downregulate the levels of P62 protein, thereby regulating the proliferation and apoptosis of CC cells, but autophagy inhibitor 3-MA can reverse these effects (Chen M. et al., 2019). Same as circMTO1, western blot results found that the combination of hsa_circ_0023404 decreased the levels of P62 and Beclin 1 protein (Guo et al., 2019). Studies have also shown that hsa_circ_0023404 enhanced chemical resistance to cisplatin in CC cells by regulating autophagy signaling. Hsa_circ_0023404 can be bound to miR-5047 directly. Knockout hsa_circ_0023404 reduced the number of invasive cells and markedly attenuated lymphatic formation. VEGFA can partially restore CC cells metastasis regulated by hsa_circ_0023404 or miR-5047, suggesting that VEGFA is an essential downstream effector of hsa_circ_0023404/miR-5047-mediated CC cell migration. Hsa_circ_0000515 silencing also inhibits cancer progression by regulating autophagy genes. For example, the expression of PCNA, MMP-9, TIMP-1, and P62 decreased, which is associated with higher expression of caspase 3, caspase 9, Beclin 1, LC3, and LC3-II/LC3-I rates (Tang et al., 2019).

#### Regulation of Other Genes Through Sponge Mechanisms

Using microarray screen CC specific circRNA, such as circ_0005576 (Ma et al., 2019),hsa_circ_0018289 (Gao et al., 2017) and circRNA-000284 (Ma et al., 2018). Target genes KIF20A, KIF20A of circ_0005576 were screened by GEPIA and Starbase database, which was extremely elevated in CC tissues. CC tissues also have sequences complementary to miR-153-3p. Downregulation of circ_0005576 in CC cell lines decreases the levels of KIF20A protein, and using the miR-153-3p inhibitor can partially retrieve the downregulation of KIF20A caused by circ_0005576 knockdown. Study disclosed that circ_0005576/miR-153-3p/KIF20A axis can encourage the development of CC, which can provide a novel understanding of the tumorigenesis of CC (Ma et al., 2019). Through bioinformatics prediction procedures, luciferase reporter assays, and RIP assays, hsa_circ_0018289 and miR-497 may interact via sponge mechanisms (Gao et al., 2017). CircRNA-000284 regulates cell proliferation and invasion via binding to miR-506 and subsequently targeting Snail-2 genes that are upregulated by circRNA-000284 (Ma et al., 2018).

Hsa_circ_0000263 (Cai et al., 2019), hsa_circ_0007534 (Rong et al., 2019), and circAMOTL1 (Ou et al., 2020a) was dramatically upregulated in CC by RT-qPCR analysis. They all promoted CC cell colony formation *in vitro*. Downregulation of circ-0000263 and circAMOTL1 could reduce tumor size *in vivo* experiments. There are direct interactions between hsa_circ_0000263 and miR-150-5p, between hsa_circ_0007534 and miR-498, and between circAMOTL1 and miR-485-5p through the sponge mechanism. Rescue experiments suggest that downregulation of hsa_circ_0007534 inhibits cell proliferation and migration by interacting miR-498/BMI-1 axis (Rong et al., 2019). The MiR-485-5p/AMOTL1 axis participates in cervical cancer progression, which was mediated by circ_AMOTL1 (Ou et al., 2020a). It was proven that hsa_circ_0000263 sponging miR-150-5p could adjust downstream factor murine double minute 4 (MDM4) expression and ultimately regulate the expression of p53 genes, revealing the important function of hsa_circ_0000263/miR-150-5p/MDM4/p53 pathways in CC (Cai et al., 2019). Circ-ITCH also performed tumor suppressor activity and affected the expression level of Forkhead Box K2 (FOXK2) by sponging miR-93-5p (Li et al., 2020).

### Encoding Cancer Proteins

Researchers have invented a pipeline that could find back-splice junctions from viral genomes to screen the circRNA presenting in HPV. They detected HPV16 circE7 from cells transformed by HPV16, preferentially located in the cytoplasm, which is related to polyribosomes. HPV16 and HPV35 circE7s are both predicted to have miRNA binding sites between HPV16 and HPV35 circE7 species, but none of them are conserved, so circE7 cannot be an miRMA sponge. RIP experiments confirmed that circE7 was m6A modified and was able to produce E7 protein in the form of heat shock regulation. Inhibiting circE7 mutations formed by DRACH motif mutations or splicing site mutations can prevent the translation of E7 proteins. Although the quantification of northern imprinting indicates that circE7 accounts for about 1–3% of the total E7 transcript and has lower transcript abundance compared to linear HPV transcript, and circE7 has a crucial impact on the function of CaSki cells transformed by HPV16. Targeting the circE7 back-splicing connection rather than targeting siRNA of linear homotypes prevents the production of E7 oncoproteins *in vitro* and decreases cellular proliferation in tumor xenografts. At the same time, circE7 only existed in the cell lines with free HPV and are found in TCGA RNA-Seq data of HPV-positive cancers (Zhao et al., 2019). These studies demonstrate that virus-derived, protein-coding circRNA have biological functions and are linked to the transformation properties of certain HPV. Results of circE7 formation may generate new understandings about how HPV can infect, lurk, and cause tumors to develop, and further studies that detect circE7 reverse joint connections may also have diagnostic significance.

### Interacting With RNA-Binding Proteins (RBPs)

QKI, a kind of RBP, can activate biogenesis and cyclization in tumorigenesis of circRNA. It was indicated that QKI could be bound to specific QKI response elements (QREs) in the flanking introns of circSLC26A4 using RIP analysis. In order to prove this, several fusion vectors with QRE mutants were constructed, and circSLC26A4 expression was detected by qRT-PCR, proving that QKI can effectively activate circSLC26A4 by interacting with two QRE in flanking introns, and they can then target miR-1287-5p/HOXA7 to impact the progression of cervical cancer (Ji et al., 2020). Another RBP, called Single-Stranded DNA Binding Protein 1 (SSBP1), interacts with hsa_circ_0072088 (circZFR). They can form an SSBP1-circZFR complex that activates the CDK2/cyclin-E1 complex to induce p-Rb phosphorylation, thereby releasing the E2F1 transcription factor. Therefore, this promotes the progression of the CC cell cycle and induces the proliferation of CC cells (Zhou et al., 2021). In another study, circCDKN2B-AS1 promoted the aerobic glycolysis of CC cells by recruiting IM3 protein, which is also a kind of RBP, to regulate the stability of the rate-limiting enzyme Hexokinase 2 (HK2) mRNA in the aerobic glycolysis pathway. Moreover, the bond between the cyclic KN2B-AS1 and IM3 proteins can be blocked by IIP, which is a synthetic inhibitory peptide, opening up a new way for the treatment of cervical cancer (Zhang et al., 2020).

## Application of CircRNA in Clinical Research of Cervical Cancer

### Impact on Clinical Severity

RT-qPCR confirmed in CC cell lines and tumor tissues the expression of circ-MYBL2 (Wang J. et al., 2019), hsa_circ_0075341 (Shao et al., 2020),hsa_circ_0001038 (Wang Y. et al., 2020), circRNA8924 (Liu et al., 2018), circ_0005576 (Ma et al., 2019), circCLK3 (Hong et al., 2019), and circ_0018289 (He et al., 2020) was significantly upregulated. Their elevation is closely related to clinical severity, including tumor size, FIGO staging, lymph node metastasis, and myometrial invasion, and this was also associated with poor prognosis in CC patients. The potential circRNAs that could be used as biomarkers in CC are shown in Table 2.

**TABLE 2:** The potential circRNAs that could be used as biomarkers in clinical application.

circRNA	miRNA	Target gene	Clinical application	Type of detection methods	References
Hsa_circ_0101996 and hsa_circ_0101119	–	–	The expression of Hsa_circ_0101996 and hsa_circ_0101119 were significantly upregulated in peripheral whole blood from CC patients	RT-qPCR and ROC analysis	Wang Y.-M. et al., 2017
circCLK3	miR-320a	FoxM1	Significantly correlated with poor tumor differentiation, advanced FIGO stages, and large depth of stromal invasion but was negatively related with OS and disease-free survival of CC patients	qRT-PCR and Kaplan–Meier Plotter analysis	Hong et al., 2019
circ_0005576	miR-153	KIF20A	Upregulated circ_0005576 was positively associated with advanced FIGO stage, lymph node metastasis, but was negatively related with OS of CC patients	qRT-PCR, Kaplan–Meier Plotter analysis, and IHC	Ma et al., 2019
circ-MYBL2	miR-361-3p	–	The expression of circ-MYBL2 was significantly upregulated and positively associated with advanced FIGO stage, larger tumor size, lymph node metastasis, and poor prognosis in CC patients	qRT-PCR and Western blotting	Wang J. et al., 2019
hsa_circ_0075341	miR-149-5p	AURKA	The expression of hsa_circ_0075341 was significantly upregulated and associated with larger tumor size, advanced FIGO stage, and lymph-node metastasis in CC patients	qRT-PCR, IHC, and Kaplan-Meier curves	Shao et al., 2020
hsa_circ_0001038	miR-337-3p	CNNM3, MACC1	Hsa_circ_0001038 highly expressed in CC tissues and was closely related to lymph node invasion and myometrial invasion	qRT-PCR, Western blotting, Kaplan-Meier curves, Fisher’s exact test, and Pearson correlation analysis	Wang Y. et al., 2020
circRNA8924	miR-518d-5p/519-5p	CBX8	The level of circRNA8924 expression was significantly correlated with tumor size, FIGO staging and myometrial invasion, but it has no correlation with patient’s age or tumor differentiation or lymph node metastasis	qRT-PCR and Western blotting	Liu et al., 2018
circ_0000388	miR-337-3p	TCF-12	The circ_0000388 expression was notably correlated with the FIGO stage, lymph node metastasis, and depth of invasion	qRT-PCR and Western blotting	Meng et al., 2020
circ_103973	miR-335	PPP6C	Higher levels of circ_103973 were correlated to a worse prognosis of CC patients	qRT-PCR	Zhu et al., 2020
circRNA_0000285	–	FUS	CircRNA_0000285 expression was significantly higher in CC tissue samples	qRT-PCR	Chen R. X. et al., 2019
Hsa_circRNA_ 101996	miR-8075	TPX2	The expression level of hsa_circRNA_101996 in CC tissues was positively correlated with TNM stage, tumor size, and lymph node metastasis	qRT-PCR and Kaplan-Meier analysis	Song et al., 2019
circular RNA_0018289	–	–	Circ_0018289 expression was positively correlated with pathological grade, tumor size, lymph node metastasis, and FIGO stage	RT-qPCR, ROC analysis, and Cox’s regression analysis	He et al., 2020
circular FoxO3a	–	–	Low serum circFoxO3a levels to be a poor prognostic factor for both OS and RFS, independent of positive lymph node metastasis	RT-qPCR, Kaplan-Meier analysis, and multivariate Cox regression analysis	Tang et al., 2020
circZFR	–	SSBP1/CDK2/cyclin E1	CircZFR was positively associated with lymphatic metastasis and was not associated with age, tumor stage, invasion depth, or vascular invasion	qRT-PCR and ROC analysis	Zhou et al., 2021

Mechanically, circ-MYBL2 binds to miR-361-3p in CC cells, and the inhibition of miR-361-3p reverses the effect of si-circ-MYBL2 on the progression of CC cells (Wang J. et al., 2019). Hsa_circ_0075341 negatively regulates miR-149-5p in CC progression. AURKA has been demonstrated to combine with miR-149-5p and is positively regulated by hsa_circ_0075341 in CC. Low miR-149-5p expression was associated with the poor overall survival (OS) rate (hazard ratio (HR) = 0.96, *P* < 0.05) of CC patients. Expression of AURKA was upregulated and also related to poor OS (*P* < 0.05) and lymph node metastasis in cervical cancer (Shao et al., 2020). The patients with higher expression of hsa_circ_0001038 can reduce OS more than those with a lower expression (*P* = 0.030). Hsa_circ_0001038 also negatively correlated with miR-337-3p expression in CC tissues. The expression of hsa_circ_0001038 in clinical tissues was also positively correlated with the expression level of metastasis-associated in colon cancer 1 (MACC1) and cyclin-M3 (CNNM3). Hsa_circ_0001038 allows miR-337-3p to release the inhibition of CNNM3 and MACC1 and can be associated with metastasis, thus promoting the growth and invasion of CC cells (Wang Y. et al., 2020). CircRNA8924 and the miR-518d-5p/519-5p family regulates CBX8 expression levels through competitive bonding, and miR-518d-5p/519-5p expression levels are thus negatively correlated with circRNA8924. Upregulation of CBX8 expression enhances malignant phenotype of CC cells (Liu et al., 2018).

Besides, upregulation of circ_0000388 expression in CC clinical samples is associated with poor pathological indicators. Circ_0000388 overexpression significantly hindered miR-337-3p expression and enhanced TCF12 expression to play a carcinogenic role in CC tissue and cells (Meng et al., 2020). High expression of circ_103973 is also related to poor prognosis in CC patients, and circ_103973 is an miR-335 sponge that directly targets PPP6C in cells (Zhu et al., 2020). The expression level of circRNA_0000285 was strongly higher than the corresponding normal tissues in CC samples. The growth and migration ability of CC cells was significantly inhibited after knockout of circRNA_0000285 *in vitro*, and the expression of FUS was also downregulated. Moreover, in nude mice, knockdown circRNA_0000285 can obviously inhibit the production and metastasis of the tumor (Chen R. X. et al., 2019). At the same time, the expression of circRNA_0000285 was raised in patients with radioresistant nasopharyngeal carcinoma compared with patients with radiosensitive nasopharyngeal carcinoma (Shuai et al., 2018). However, whether it relates to the radiosensitivity of CC needs further exploration. CircRNA_101996 obtained using circRNA microarrays is positively correlated with CC staging and negatively correlated with OS rate (*P* = 0.032), promoting CC progression through hsa_circRNA_101996/miR-8075/TPX2 networks (Song et al., 2019).

### As a Diagnostic Biomarker

The expression levels of five circRNAs (hsa_circ_0101996, hsa_circ_0104649, hsa_circ_0104443, and hsa_circ_0101119) are markedly increased in the peripheral blood of 87 CC patients and 55 healthy controls. It is speculated that the expression of circRNA of whole blood in CC patients may have specific changes. We used the Receiver Operating Characteristic (ROC) curve to plot the expression level of circRNA in CC tissue and its paired ANT to evaluate the diagnostic value of circRNA. The area under the ROC curve (AUC) of hsa_circ_0101996 and hsa_circ_0101119 in peripheral whole blood was 0.906 and 0.887, respectively. The combined AUC increased to 0.964 (Wang Y.-M. et al., 2017). Low expression of serum circFoxO3a was significantly associated with positive lymph node metastasis (*P* = 0.008) and deeper stromal invasion depth (*P* = 0.005). CC patients with lower expression of serum circFoxO3a negatively correlated with OS rate and recurrence-free survival (RFS) (Tang et al., 2020). Hsa_circ_0101996, hsa_circ_0101119, and circFoxO3a in peripheral whole blood of CC patients can be combined as diagnostic biomarkers in routine clinical diagnosis. ROC analysis showed that the AUC of circ_0018289 was 0.907 (He et al., 2020), the AUC of hsa_circ_0107593 was 0.869 (Liao et al., 2020), and the AUC of circZFR was 0.88, which could significantly separate tumor tissue from ANT. Moreover, the clinical pathological features of 40 patients with CC revealed a positive correlation between circZFR expression and lymphatic metastasis, Ki67 values, and squamous cell carcinoma antigen (SCC Ag) value (Zhou et al., 2021). So circ_0018289, hsa_circ_0107593, and circZFR can also serve as potential disease monitoring biomarkers of CC.

### As a Drug Target

Some preliminary studies have demonstrated that Spatholobi Caulis tannin (SCT), as a traditional Chinese medicine, has anti-cancer properties. A three-dimensional microfluidic chip demonstrates that SCT can reduce the survival rate of Hela cells and regulate the cell cycle, and it has been shown to affect pathogenic proteins by molecular docking technology (Wang et al., 2018). Differential genes of cervical cancer were screened by the TCGA database and GEO and KEGG analysis. Using molecular docking studies and other bioinformatic analyses predicted SCT target proteins to further identify circRNAs associated with SCT target genes. The expression levels of circFANCB, circE2F3, circATR, and circBLM gradually increased following the decrease of drug concentration in HeLa cells. RegRNA analysis provides the following projections: circFANCB combined with hsa-miR-4692 and circE2F3 combine with hsa-miR-5006-3p, hsa-miR-3960, hsa-miR-4739, hsa-miR-4459, hsa-miR-211-5p, and hsa-miR-4632. SCT regulates circRNAs associated with CC to promote apoptosis and inhibit the proliferation of cells. Therefore, SCT can be used as a booster for the treatment of CC (Wang N. et al., 2019).

## Conclusion

At present, the era of “precision medicine” has come; “precision medicine” is used to provide cancer patients with more individualized diagnosis, treatment, and follow-up, and improving efficacy and quality of life and reduce the treatment-related toxicity response has thus become possible. CircRNA is one of the hot topics in the study of transcriptomes. Because of its rich, stable, and specific expression, circRNA has a broad clinical application prospect and has the potential to be a molecular marker for tumor diagnosis and prognosis as well as a therapeutic target. Nevertheless, the current research on the mechanism of circRNA action in cervical cancer is not deep enough, mostly limited to the sponge mechanism between circRNA and miRNA, and its regulation needs further exploration and in-depth research.

## Author Contributions

JL: writing manuscripts and collecting literature. HZ: collecting literature and modifying manuscripts. LF: proposing amendments. TX: designing the content of the article and proposing amendments. All authors contributed to the article and approved the submitted version.

## Conflict of Interest

The authors declare that the research was conducted in the absence of any commercial or financial relationships that could be construed as a potential conflict of interest.

## Abbreviations

circRNA: circular RNA
RBPs: RNA binding proteins
CC: cervical cancer
ceRNA: competing endogenous RNA
CDR1as: cerebellar degeneration-related protein 1 transcript
Sry: sex-determining region Y
HCC: human hepatocellular carcinoma
HESCs: human embryonic stem cells
RIP: RNA binding protein immunoprecipitation
HSEs: hematopoietic stem cells
LT-HSC: long-term hematopoietic stem cells
IFN: interferon
FISH: fluorescence *in situ* hybridization
EIciRNAs: exon-intron circRNA
snRNP: small nuclear ribonucleoproteins
IRES: internal ribosome entry site
m6a: modified n6- methyladenosine
PPI: protein-protein interaction
GEO: Gene Expression Omnibus
CGA: Cancer Genome Atlas
ANT: adjacent non-tumor tissue
CSCC: cervical squamous cell carcinoma
DE: differentially express
KEGG: Kyoto Encyclopedia of Genes and Genomes
GO: Gene Ontology
HPV: human papillomavirus
PI3K: phosphatidylinositol 3-kinase
TGF- β: transforming growth factor-beta
MAPK: mitogen-activated protein kinase
EMT: epithelial-mesenchymal transition
HOXA1: homeobox a1
RNA-FISH: RNA fluorescence *in situ* hybridization
QREs: QKI response elements
EGFR: epidermal growth factor receptor
ATG: autophagy-related genes
MDM4: murine double minute 4
FOXK2: Forkhead Box K2
SSBP1: Single-Stranded DNA Binding Protein 1
OS: overall survival
HR: hazard ratio
MACC1: metastasis-associated in colon cancer 1
CNNM3: cyclin-M3
ROC: receiver operating characteristic
AUC: area under curve
RFS: recurrence-free survival
SCC Ag: squamous cell carcinoma antigen
SCT: Spatholobi Caulis tannin.
